# Nierenerkrankungen bei Diabetes mellitus Typ 2: Überblick und Implementierung der Leitlinien, Positionspapiere und Praxisempfehlungen zur Diagnostik und Überwachung

**DOI:** 10.1007/s00108-023-01610-1

**Published:** 2023-11-13

**Authors:** Monika Kellerer, Christoph Wanner

**Affiliations:** 1 Klinik für Innere Medizin 1, Marienhospital Stuttgart, Böheimstr. 37, 70199 Stuttgart, Deutschland; 2 Medizinische Klinik und Poliklinik mit Schwerpunkt Nephrologie, Universitätsklinikum Würzburg, Oberdürrbacher Str. 6, 97080 Würzburg, Deutschland

**Keywords:** Albuminurie, Geschätzte glomeruläre Filtrationsrate, Niereninsuffizienz, Albumin-Kreatinin-Verhältnis im Urin, Nephrologische Mitbetreuung, Albuminuria, Glomerular filtration rate, estimated, Renal insufficiency, Urine albumin to creatinine ratio, Referral/nephrology

## Abstract

**Hintergrund:**

Nationale und internationale Fachgesellschaften publizieren Leitlinien zur Diagnostik und Verlaufsbeobachtung einer chronischen Nierenerkrankung bei Menschen mit Diabetes mellitus Typ 2. Über die Kongruenz und Implementierung dieser Publikationen im klinischen Alltag wird jedoch selten berichtet.

**Ziel der Arbeit:**

Diese Arbeit bietet einen Überblick über die Empfehlungen als Ausdruck des globalen Wissensstands und eruiert deren Umsetzung im deutschen Praxisalltag. Dazu wurde eine repräsentative Befragung erhoben.

**Material und Methoden:**

Aktuelle Leitlinien wurden in Bezug auf Kongruenz der folgenden Aspekte verglichen: diagnostische Parameter, Testfrequenz und Empfehlungen zur nephrologischen Mitbetreuung. Die Ergebnisse wurden im nächsten Schritt mit den Antworten aus der Befragung verglichen. So war es möglich, die Umsetzung im deutschen Praxisalltag einzuschätzen.

**Ergebnisse:**

Laut Empfehlungen sollten die geschätzte glomeruläre Filtrationsrate (eGFR) und das Albumin-Kreatinin-Verhältnis im Urin mindestens 1‑mal pro Jahr bei allen Menschen mit Diabetes mellitus Typ 2 bestimmt werden. Bei höhergradiger Niereninsuffizienz (ab Kidney-Disease:Improving-Global-Outcomes[KDIGO]-Stadium 3b mit eGFR < 45 ml/min/1,73 m^2^) bzw. Albuminurie (ab Stadium A2) sind eine häufigere Bestimmung sowie die nephrologische Mitbetreuung empfehlenswert; hier werden jedoch unterschiedliche Schwellenwerte und Frequenzen empfohlen. In der Auswertung der Fragebögen wurde die jährliche Bestimmung der eGFR in 96,5 % aller Fragebögen positiv beantwortet, die Bestimmung der Albuminurie in 77,2 %. Eine eGFR-getriggerte nephrologische Mitbetreuung wird von 19,6 % der nichtnephrologischen Praxen umgesetzt; die Albuminurie-getriggerte Mitbetreuung erfolgt in der Mehrzahl der Fälle.

**Schlussfolgerungen:**

Die Messung der eGFR ist als Standard in Deutschland etabliert. Verbesserungspotenzial ergibt sich bei Albuminuriemessung, Häufigkeit der Testung und Zeitpunkt der nephrologischen Konsultation. Die interdisziplinäre Zusammenarbeit wird von allen Leitlinien betont.

## Abstract (en)

**Background:**

National and international medical societies have published guidelines and recommendations pertaining to the diagnostics and monitoring of chronic kidney disease in patients with type 2 diabetes mellitus. Consistency and implementation in daily clinical practice are rarely reported.

**Objective:**

This article provides an overview on recommendations as a reflection of the global state of the art and assesses the implementation in daily practice in Germany, which was collected via a representative questionnaire.

**Material and methods:**

The current guidelines were compared with respect to the consistency of parameters, frequency of testing and recommendations for nephrological referrals. The results were then compared with the survey responses to estimate the level of their implementation in daily practice in Germany.

**Results:**

According to the recommendations the estimated glomerular filtration rate (eGFR) and the urine albumin to creatinine ratio (UACR) should be tested at least once per year in all patients with type 2 diabetes. In cases of more severe kidney impairment (above Kidney Disease:Improving Global Outcomes, KDIGO, stage 3b with eGFR < 45 ml/min/1,73 m^2^) or albuminuria (from stage A2), more frequent measurements and nephrological referrals are recommended; however, different threshold values and frequencies are recommended. The responses from the questionnaires indicate that eGFR is tested annually in 96.5% of all cases and albuminuria is tested in 77.2% of cases. An eGRF triggered referral to a nephrologist is implemented by 19.6% of all nonnephrological practitioners, albuminuria triggered referrals are implemented in the majority of cases.

**Conclusion:**

Measurement of eGFR is the established standard in Germany. Potential improvement was found in albumin measurement, the frequency of testing and the time point for nephrological consultation. All guidelines emphasize the benefits of interdisciplinary cooperation.

Diabetes mellitus ist eine stille Epidemie, unter der im Jahr 2021 geschätzt 537 Mio. Menschen weltweit litten. Es wird erwartet, dass sich diese Zahl bis zum Jahr 2045 auf etwa 784 Mio. Menschen erhöht, von denen wiederum 40 % eine chronische Nierenerkrankung, einschließlich Dialyse und Transplantation, als Folge des Diabetes mellitus entwickeln werden [1]. Dies verdeutlicht die Notwendigkeit, sich intensiv mit der frühen Diagnostik und der Verlaufsbeobachtung diabetesassoziierter Nierenschädigungen zu beschäftigen, um frühzeitig zielgerichtete Therapien einleiten und adaptieren zu können.

Die Stadieneinteilung der chronischen Nierenerkrankung im Rahmen des Diabetes mellitus folgt standardmäßig den Kidney-Disease:Improving-Global-Outcomes(KDIGO)-Empfehlungen, die bereits 2013 publiziert [2] und unverändert in die Überarbeitung aus dem Jahr 2022 [3] sowie in die Leitlinien der American Diabetes Association (ADA; [5]) und der European Society of Cardiology (ESC) übernommen wurden [6]. Kernstücke sind die Bestimmung der geschätzten glomerulären Filtrationsrate (eGFR) und der Albuminurie, für die das Albumin-Kreatinin-Verhältnis im Urin („urine albumin-to-creatinine ratio“ [UACR]) als internationaler Goldstandard etabliert ist [2, 3]. Aus beiden Werten können das Ausmaß der Nierengewebeschädigung und die Einschränkung der Nierenfunktion ermittelt sowie die daraus resultierende Häufigkeit der Untersuchungen pro Jahr abgeleitet werden [2, 3].

Das Ausmaß der Albuminurie sowie die Veränderung der eGFR gelten als unabhängige Risikofaktoren

Wichtig ist zudem, dass das Ausmaß der Albuminurie sowie die Veränderung der eGFR als unabhängige Risikofaktoren interpretiert werden müssen. Damit reicht die Bestimmung nur eines Faktors nicht aus. Vielmehr verbessert eine Bestimmung beider Parameter zwangsläufig die Zielgenauigkeit der einzuleitenden Maßnahmen [4].

Ziel des vorliegenden Beitrags ist es, einen Überblick über die Empfehlungen führender nationaler und internationaler Leitlinien in Bezug auf die Diagnostik einer chronischen Nierenerkrankung bei Diabetes mellitus Typ 2 als Ausdruck des globalen Wissensstands zu erarbeiten. Zudem soll deren Umsetzung im deutschen Praxisalltag auf Basis einer repräsentativen Befragung beschrieben werden.

## Auswahl relevanter Leitlinien, Positionspapiere und Praxisempfehlungen

Folgende Publikationen wurden vom Autorenteam als relevant erachtet^1^:

- Kidney Disease: Improving Global Outcomes (KDIGO) Diabetes Work Group. KDIGO 2022 Clinical Practice Guideline for Diabetes Management in Chronic Kidney Disease [3]
- Consensus Report by the American Diabetes Association (ADA) and Kidney Disease: Improving Global Outcomes (KDIGO), 2022 [11]
- American Diabetes Association (ADA). Standards of Medical Care in Diabetes 2023 [5]
- European Society of Cardiology (ESC) in collaboration with the European Association for the Study of Diabetes (EASD). Guidelines on diabetes, pre-diabetes, and cardiovascular diseases, 2019 [6]
- Deutsche Gesellschaft für Allgemeinmedizin und Familienmedizin (DEGAM). S3-Leitlinie Versorgung von Patienten mit chronischer, nichtdialysepflichtiger Nierenerkrankung in der Hausarztpraxis, 2019 [7]
- Deutsche Diabetes Gesellschaft (DDG). DDG Praxisempfehlung Nephropathie bei Diabetes, 2022 [8]
- Nationale VersorgungsLeitlinie (NVL). Typ-2-Diabetes, Langfassung, 2023 [9]

Diese aktuellen Leitlinien und Positionierungen der verschiedenen Fachgesellschaften wurden miteinander auf Kongruenz hinsichtlich der Diagnostik einer diabetesassoziierten Nephropathie verglichen, insbesondere bezüglich der empfohlenen Parameter, der Frequenz der Testung sowie der Empfehlungen zur nephrologischen Mitbetreuung. Die Ergebnisse wurden im nächsten Schritt mit den Antworten aus der Befragung verglichen und so eine Einschätzung der Umsetzung im deutschen Praxisalltag erarbeitet.

## Methodik der repräsentativen Befragung

Im Rahmen der Umfrage wurden insgesamt 24.530 Ärztinnen und Ärzte in Deutschland per E‑Mail und/oder Brief kontaktiert. Der Fokus lag auf denjenigen Facharztgruppen, die mit hoher Wahrscheinlichkeit in die Versorgung von Patienten mit Diabetes mellitus Typ 2 eingebunden sind.

Antworten konnten entweder internetbasiert über ein Smart-Survey-Tool eingegeben oder als Fax bzw. Brief zurückgesendet werden. Es wurde keine Aufwandsentschädigung angeboten. Eine Auswertung der Antworten anhand bestimmter Charakteristika, beispielsweise Fachrichtung oder Zusatzbezeichnungen, war möglich.

## Ergebnisse

### Kongruenz der Leitlinien

In Tab. 1 findet sich ein Überblick über die empfohlenen Parameter sowie deren Erhebungsfrequenz in den verschiedenen Leitlinien. Allgemein wird darauf hingewiesen, dass für die Definition der chronischen Nierenerkrankung ein Bestätigungstest nach 3 Monaten empfehlenswert ist.

**Table 1:** 

	KDIGO, 2022 [3]; ADA-KDIGO Consensus, 2022 [11]	ADA, 2023 [5]	ESC/EASD, 2019 [6]	DEGAM, 2019 [7]	DDG, 2022 [8]	NVL Typ-2-Diabetes, 2023 [9]
**Parameter**						
*eGFR*	Ja	Ja	Ja	Ja	Ja	Ja
*Albuminurie*	Ja	Ja	Ja	Nur bei besonderen Risikopatienten	Ja	Ja
**Häufigkeit**						
*Alle Diabetes-mellitus-Typ‑2-Erkrankungen ab Diagnose*	1‑mal/Jahr	1‑mal/Jahr	1‑mal/Jahr	1‑mal/Jahr	1‑mal/Jahr	1‑mal/Jahr
*Unklares oder pathologisches Ergebnis*	Je nach Schweregrad, bis > 4-mal/Jahr	Je nach Schweregrad, bis > 4-mal/Jahr	k. A.	Je nach Schweregrad, bis zu alle 3 Monate	Alle 3 Monate	k. A.

*ADA* American Diabetes Association, *DDG* Deutsche Diabetes Gesellschaft, *DEGAM* Deutsche Gesellschaft für Allgemeinmedizin und Familienmedizin, *EASD* European Association for the Study of Diabetes, *eGFR* geschätzte glomeruläre Filtrationsrate, *ESC* European Society of Cardiology, *k.* *A.* keine Angabe, *KDIGO* Kidney Disease: Improving Global Outcomes, *NVL* Nationale VersorgungsLeitlinie

Die empfohlenen Parameter zu Diagnostik und Monitoring sind – mit Ausnahme der DEGAM-Leitlinie – über alle Leitlinien hinweg eindeutig und bestehen in der Bestimmung der eGFR und der UACR. Bezüglich der UACR spricht sich die DEGAM für eine individuell zu prüfende Bestimmung in spezifischen Risikogruppen aus, das heißt bei Menschen mit Typ-2-Diabetes, die eine schlecht kontrollierte Plasmaglukose bzw. Bluthochdruck haben, gegebenenfalls für Letzteres noch keinen Angiotensin-converting-enzyme(ACE)-Hemmer bzw. Angiotensin-II-Rezeptor-Subtyp-1(AT1)-Antagonisten erhalten und die zugleich zu einer Therapieeskalation bereit sind, wenn sie vom Vorhandensein des zusätzlichen Risikofaktors „Albuminurie“ wissen [7].

Konsens besteht ebenfalls hinsichtlich der jährlichen Testung aller Menschen mit Typ-2-Diabetes. Hinsichtlich der Frequenz bei Menschen mit unklarem und/oder pathologischem Ergebnis ist eine geringe Heterogenität vorhanden. Die Leitlinie der DEGAM empfiehlt, die Frequenz individuell mit dem Patienten abzustimmen und nicht einem festen Intervall zu folgen. Die DEGAM-Empfehlungen orientieren sich hier an der Leitlinie des National Institute for Health and Care Excellence (NICE; [10]) und weichen aus „pragmatischen Gründen“ [7] von den KDIGO-Empfehlungen ab [3]. Darüber hinaus empfiehlt die DEGAM, dass bei asymptomatischen Erwachsenen ohne weitere Risikofaktoren für eine chronische Nierenerkrankung kein regelmäßiges Screening durchgeführt werden sollte.

Die NVL Typ-2-Diabetes hat ihren Schwerpunkt in der differenzierten Bewertung der Diagnostik und Therapie des Typ-2-Diabetes. Diesem Schwerpunkt folgend unterstützt die 2023 publizierte Langfassung die Untersuchung mittels eGFR und UACR im Rahmen einer integrierten Risikoeinschätzung, weist jedoch auch auf die Unterschiede in der Bewertung dieser beiden Parameter durch die beteiligten Fachgesellschaften hin.

Zur nephrologischen Mitbetreuung finden sich Empfehlungen in den Leitlinien der ADA [5] und im ADA-KDIGO Consensus Report [11]. Während die ADA-Leitlinie eine nephrologische Vorstellung aller Patienten mit einer eGFR < 30 ml/min/1,73 m^2^ empfiehlt [5], ergänzt der ADA-KDIGO Consensus Report auch die nephrologische Vorstellung aller Menschen mit Albuminurie Stadium A3 (UACR ≥ 300 mg/g) unabhängig von der eGFR [11].

Die DEGAM betont, dass Prävention und Palliativversorgung genuin hausärztliche Tätigkeiten seien

Eine sehr detaillierte Darstellung ist in der Leitlinie der DEGAM zu finden, die einerseits zwischen einer einmaligen Vorstellung und der kontinuierlichen Mitbetreuung durch Nephrologen unterscheidet und andererseits eine umfangreiche Übersicht über die Empfehlungen anderer Leitlinien wie der Deutschen Gesellschaft für Nephrologie (DGfN) enthält. Hier finden sich auch weitere Parameter, die eine nephrologische Mitbehandlung empfehlenswert erscheinen lassen, so etwa

- jede erstmals festgestellte eGFR unter 30 ml/min/1,73 m^2^ und
- jede Kombination einer eGFR zwischen 30 und 60 ml/min/1,73 m^2^ mit einer
  - persistierenden, nicht urologisch erklärbaren Hämaturie (Stärke 2+),
  - Albuminurie in Stadium ≥ A2 oder
  - refraktären Hypertonie unter ≥ 3 Blutdruckmedikamenten.

Die Leitlinie der DEGAM betont jedoch, dass Prävention und Palliativversorgung genuin hausärztliche Tätigkeiten seien und im Regelfall von dieser Fachgruppe erbracht werden [7].

Die Praxisempfehlung der DDG hebt den Wert einer frühzeitigen, engen und patientenzentrierten Kooperation zwischen Allgemeinmedizin, Diabetologie, Nephrologie und Kardiologie hervor und erachtet eine nephrologische Vorstellung bei Erreichen eines Niereninsuffizienzstadiums 3a nach KDIGO (eGFR 45–59 ml/min/1,73 m^2^) für alle Menschen mit Diabetes bzw. bei Erreichen des Stadiums 3b (eGFR 30–44 ml/min/1,73 m^2^) für diejenigen über 75 Jahre als notwendig [8].

Die Leitlinien der ESC [6] und die NVL Typ-2-Diabetes [9] geben zu dieser Frage keine Empfehlung ab.

Aus den oben genannten Leitlinien und Praxisempfehlungen lassen sich folgende grundlegende Gemeinsamkeiten herausarbeiten:

- Zu bestimmende Parameter sind die eGFR und UACR. Beide sollten mindestens 1‑mal pro Jahr bei allen Menschen mit Typ-2-Diabetes bestimmt werden.
- Bei höhergradiger Niereninsuffizienz (ab KDIGO-Stadium 3b mit eGFR < 45 ml/min/1,73 m^2^) bzw. Albuminurie (ab Stadium A2) ist eine häufigere Bestimmung sinnvoll; hier werden jedoch unterschiedliche Schwellenwerte und Frequenzen empfohlen.
- Die nephrologische Vorstellung bzw. Mitbetreuung sollte ebenfalls bei höhergradiger Niereninsuffizienz bzw. Albuminurie laut der Mehrheit der Leitlinien erwogen werden, jedoch ebenfalls ohne kongruente Schwellenwerte.

Diese grundlegenden Eckpfeiler in der Diagnostik und Verlaufsbeobachtung von Menschen mit Typ-2-Diabetes wurden mit den Ergebnissen einer fragebogengestützten Erhebung verglichen, deren erste Ergebnisse wir bereits 2022 publiziert haben [12].

### Charakterisierung der Umfrageteilnehmer

Von den ausgesandten 24.530 Fragebögen erhielten wir insgesamt 1626 ausgefüllt zurück. Führende Berufsgruppe waren Fachärztinnen und Fachärzte (FÄ) für Allgemeinmedizin (*N* = 1026; 63,1 %), gefolgt von FÄ mit Zusatzbezeichnung Diabetologie (*N* = 316; 19,4 %). FÄ für Nephrologie (*N* = 163) bzw. Kardiologie (*N* = 121) trugen mit 10,0 % bzw. 7,4 % der Einsendungen bei. Die größte Gruppe innerhalb der Diabetologen wiederum bildeten die FÄ für Innere Medizin mit 53 % (*N* = 169; [12]).

### Relevante Leitlinien für den Praxisalltag

**Figure 1:**
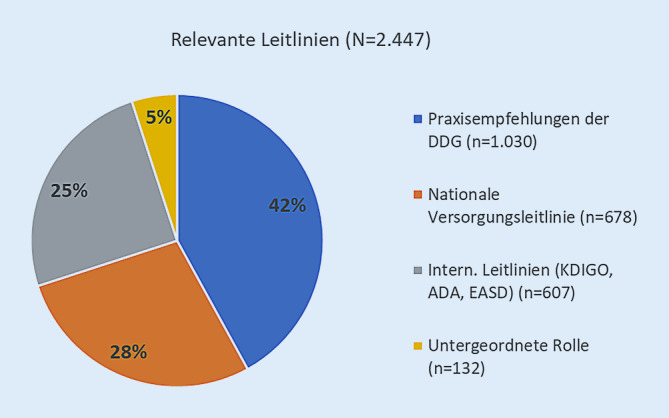

In Abb. 1 sind Leitlinien gezeigt, die als wichtig erachtet wurden. 95 % aller Befragten gaben mindestens eine Leitlinie als relevant für ihre medizinische Diagnostik an. Überwiegend wurden dabei deutschsprachige Leitlinien genannt. Internationale, sprich englischsprachige Leitlinien wurden seltener genannt. Lediglich 5 % gaben an, dass Leitlinien eine untergeordnete Rolle für ihre Entscheidung spielen.

### Umsetzung der Leitlinienempfehlungen

Legt man die Umfrageergebnisse zugrunde, sind die Diagnostik und Verlaufsbeobachtung an den Prinzipien der DDG-, NVL- und KDIGO/ADA-Empfehlungen zu messen. Gemeinsames Element dieser Empfehlungen ist die mindestens jährliche Bestimmung von eGFR und UACR bei allen Menschen mit Typ-2-Diabetes.

**Figure 2:**
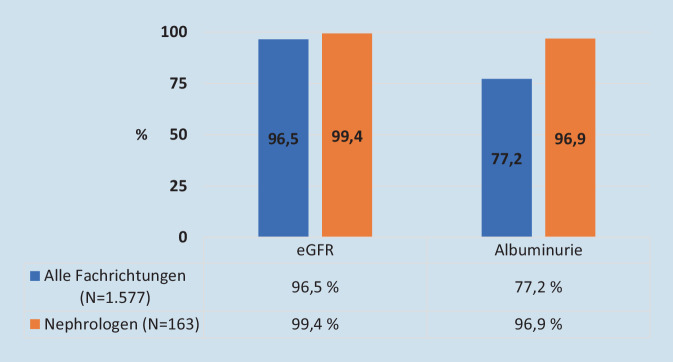

Die Bestimmung der eGFR wird in 1522 Antworten genannt (96,5 % aller Fragebögen mit einer Antwort zu dieser Frage), die Bestimmung der Albuminurie in 1218 Antworten (77,2 %). Eine deutlich häufigere Bestimmung beider Werte findet sich bei den Nephrologen, die die Bestimmung der eGFR in 162 (99,4 %) und die der Albuminurie in 158 (96,9 %) Fällen positiv beantworteten (Abb. 2).

**Figure 3:**
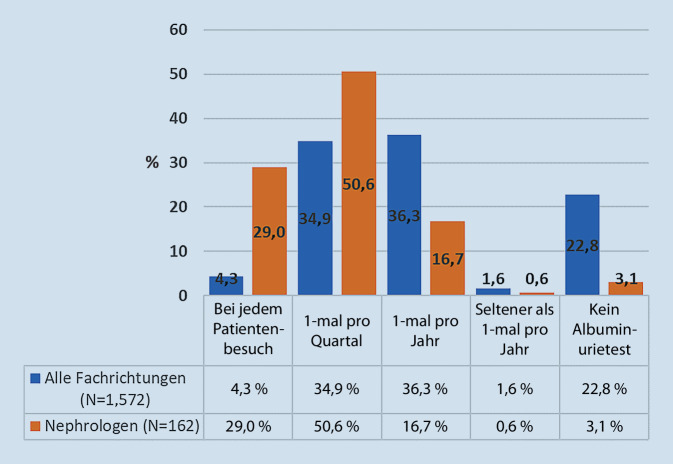

Ähnliche Ergebnisse erbrachte die Auswertung der Frage nach der Testhäufigkeit. Hier waren insbesondere die Angaben zur Häufigkeit der Albuminurietestung unterschiedlich (Abb. 3).

Insgesamt erfüllen etwa drei Viertel aller Antworten die Mindestanforderungen an die Frequenz der Albuminurietestung (mindestens 1‑mal pro Jahr). Deutlich weniger als die Hälfte erachtet eine höhere Frequenz als notwendig und weicht in diesem Punkt recht klar von den Leitlinienempfehlungen ab. Auch im Bereich der Albuminurietestung deuten die Zahlen für die nephrologischen Praxen eine deutlich höhere Umsetzung der Leitlinienempfehlungen an. Bemerkenswert ist der relativ hohe Anteil der Antwortenden, die gar keine Albuminurietestung angeben (22,8 % aller Antworten gegenüber 3,1 % der nephrologischen Praxen).

Gefragt nach den Gründen, die gegen eine regelmäßigere Urintestung in der eigenen Praxis sprechen, antworteten 34,1 % (*N* = 538) mit „fehlende zusätzliche Erstattung“ gefolgt von 23,8 % mit der Antwort „schlechte Praktikabilität, insbesondere bei älteren Menschen“ (*N* = 376).

Die Frage, ob Patienten mit Typ-2-Diabetes und chronischer Nierenerkrankung in der eigenen Praxis behandelt werden, beantworteten 45,7 % der Nichtnephrologen mit „Ja“ (*N* = 614). Die Schwellenwerte für eine Überweisung sind in Tab. 2 für die einzelnen Parameter getrennt nach nephrologischer Praxis vs. nichtnephrologischer Praxis dargestellt.

**Table 2:** 

**Parameter**	**Schwelle**	**Nephrologie (*N*** **=** **117)**	**Andere Fachrichtungen (*N*** **=** **422)**
Serumkreatinin	> 1,3 mg/dl	84 (71,8 %)	87 (20,6 %)
	> 1,5 mg/dl	31 (26,5 %)	189 (44,8 %)
	> 2,0 mg/dl	2 (1,7 %)	146 (34,6 %)
**Parameter**	**Schwelle**	**Nephrologie (*N*** **=** **129)**	**Andere Fachrichtungen (*N*** **=** **632)**
eGFR	< 30 ml/min/1,73 m^2^	3 (2,3 %)	171 (27,1 %)
	< 45 ml/min/1,73 m^2^	33 (25,6 %)	337 (53,3 %)
	*<* *60* *ml/min/1,73* *m*^*2*^	93 (72,1 %)	124 (19,6 %)
**Parameter**	**Schwelle**	**Nephrologie (*N*** **=** **119)**	**Andere Fachrichtungen (*N*** **=** **390)**
Albuminurie	> 30 mg	64 (53,8 %)	99 (25,4 %)
	> 100 mg	34 (28,6 %)	204 (52,3 %)
	> 200 mg	10 (8,4 %)	57 (14,6 %)
	*>* *300* *mg*	11 (9,2 %)	30 (7,7 %)

*Kursive* Hervorhebungen reflektieren die Schwellenwerte der DDG in Bezug auf die eGFR (ab KDIGO-Stadium 3a) bzw. des ADA-KDIGO Consensus Report hinsichtlich der Albuminurie (> 300 mg, unabhängig vom Stadium der Niereninsuffizienz)

*ADA* American Diabetes Association, *DDG* Deutsche Diabetes Gesellschaft, *eGFR* geschätzte glomeruläre Filtrationsrate, *KDIGO* Kidney Disease: Improving Global Outcomes

Insgesamt lassen diese Daten folgende Tendenzen erkennen:

- Nephrologische Praxen wünschen sich eine deutlich frühere Einbindung, als von den nichtnephrologischen Praxen als erforderlich erachtet.
- Die Empfehlung der DDG in Bezug auf eine eGFR-getriggerte Mitbetreuung durch eine nephrologische Praxis (KDIGO-Stadium 3a; 45–59 ml/min/1,73 m^2^) wird nur von 19,6 % der befragten nichtnephrologischen Praxen umgesetzt. In diesem Punkt ist die Einschätzung der Nephrologen nach Überweisung zu diesem Zeitpunkt mit 72,1 % deutlich leitlinienkonformer.
- Die Mindesterwartung des ADA-KDIGO Consensus Report in Bezug auf eine albuminuriegetriggerte nephrologische Einbindung (UACR > 300 mg/g; Stadium A3) ist in der Mehrzahl der Fälle erfüllt. Aber auch hier wünschen sich die Nephrologen die Möglichkeit der Mitbetreuung bereits bei niedrigeren Werten.

## Diskussion

Wir legen hier den zweiten Teil einer fragebogengestützten Auswertung der Versorgungsqualität bei Menschen mit Typ-2-Diabetes und chronischer Nierenerkrankung in Deutschland vor. Die Arbeit knüpft an eine Auswertung an, aus der wir eine noch unzureichende Implementierung der UACR als Routinetestparameter ableiten konnten; die möglichen Gründe hierfür haben wir diskutiert [12]. Jetzige Schwerpunkte sind die Kongruenz relevanter Leitlinien und Praxisempfehlungen hinsichtlich des Screenings auf chronische Nierenerkrankungen bei Menschen mit Typ-2-Diabetes sowie die Umsetzung der Empfehlungen zur nephrologischen Mitbetreuung im Versorgungsalltag.

Zwischen der DDG-Praxisempfehlung und den KDIGO/ADA-Standards besteht eine hohe Übereinstimmung

Fast alle Antwortenden (95 %) gaben an, sich an Leitlinienempfehlungen zu orientieren. Dies spiegelt die Relevanz von Leitlinien und Praxisempfehlungen im Versorgungsalltag wider. Dabei wurden mit der DDG-Praxisempfehlung sowie der NVL Typ-2-Diabetes in erster Linie deutschsprachige Leitlinien als relevant angesehen; internationale Leitlinien, beispielsweise von KDIGO, ADA und EASD, wurden deutlich seltener genannt. Zum Zeitpunkt der eigentlichen Befragung war weder die überarbeitete KDIGO-Leitlinie von 2022 noch das gemeinsame Positionspapier von KDIGO und ADA aus dem Jahr 2022 publiziert.

Aus Tab. 1 ist eine hohe Übereinstimmung zwischen der Praxisempfehlung der DDG und den Standards von KDIGO und ADA ersichtlich. In allen drei Leitlinien (DDG; KDIGO, ADA) wird die parallele Bestimmung von eGFR und UACR als Parameter zur Diagnostik und Verlaufsbeobachtung empfohlen. Ebenfalls nahezu deckungsgleich sind die Empfehlungen zur häufigeren Bestimmung der Werte bei höhergradiger Niereninsuffizienz und/oder Albuminurie. Da etwa 80–90 % aller Menschen mit Typ-2-Diabetes in Deutschland gleichzeitig einen Hypertonus aufweisen [13], sind die DEGAM-Empfehlungen zur Bestimmung der UACR nur bei bestimmten Risikogruppen [7] fast analog einzuordnen.

Eine Diskrepanz ergibt sich aus dem Vergleich der Empfehlungen zur eGFR- und Albuminuriebestimmung mit den Angaben in den Fragebögen zur praktischen Umsetzung. Mit über 95 % diagnostizieren und überwachen fast alle antwortenden Praxen die Nierenfunktion leitliniengerecht mit der eGFR. Diese Ergebnisse sind nahezu deckungsgleich mit den Angaben aus den deutschen Patientenregistern „Diabetes-Patienten-Verlaufsdokumentation“ und „Diabetes-Versorgungs-Evaluation“ [14]. Obwohl alle Leitlinien klar den klinischen Wert der Bestimmung beider Parameter – eGFR und Albuminurie – hervorheben, gibt es jedoch mit 77,2 % aller Antworten deutlich abfallende Werte für die Bestimmung der Albuminurie. Über 95 % der nephrologischen Praxen messen regelmäßig beide Werte und implementieren damit die Leitlinienempfehlungen deutlich stringenter. Als Argument gegen die Messung der UACR im klinischen Praxisalltag wurde dabei insbesondere die „fehlende zusätzliche Erstattung“ genannt, gefolgt von „schlechter Praktikabilität, insbesondere bei älteren Menschen“. Hinsichtlich dieser Gründe wäre in jeder Praxis zu überlegen, ob sich nicht doch Lösungen finden lassen, die diese nachvollziehbaren Herausforderungen im Sinne einer leitlinienkonformen Diagnostik überwinden. Bezüglich der fehlenden Erstattung wäre anzumerken, dass für einen manifesten Diabetes mellitus die Laborbefreiungsziffer 32022 gilt, die neben Glukose, Kreatinin und Hämoglobin A_1c_ auch einen Test auf Mikroalbumin im Urin (Einheitlicher Bewertungsmaßstab [EBM] 32135) abdeckt [15].

Aus unserer Umfrage geht weiter hervor, dass etwa die Hälfte aller antwortenden nichtnephrologischen Praxen (45,7 %) ihre Patienten mit Typ-2-Diabetes und chronischer Nierenerkrankung allein behandelt, sprich ohne nephrologische Konsultation. Dieser Ansatz spiegelt sich auch in den Antworten wider, ab welchen Schwellenwerten die nichtnephrologischen Praxen ihre Patienten beim Nephrologen vorstellen. Hier liegen die Einschätzung der Nephrologen und der nichtnephrologischen Fachrichtungen recht weit auseinander.

Gemessen an der DDG-Empfehlung überweist ein Viertel (27,1 %) der Nichtnephrologen die Patienten erst bei einem eGFR-Wert unter 30 ml/min/1,73 m^2^ weiter. Wie viele Praxen die DDG-Empfehlung einer Überweisung von unter 75-jährigen Patienten mit KDIGO-Stadium 3a (45–59 ml/min/1,73 m^2^) beachten, lässt sich aus den vorliegenden Zahlen nicht ermitteln. In Bezug auf die Albuminurie zeigt sich eine deutlich größere Sensibilität zur Überweisung. Hier zeigen die Antworten eine gute Implementierung der DDG-Empfehlungen, selbst wenn sich auch hier die Nephrologen eine frühere Mitbetreuung wünschen würden.

Als Limitation ist zu werten, dass angesichts einer Rücklaufquote unseres Fragebogens von 6,6 % eventuell bevorzugt Praxen geantwortet haben, bei denen ein größeres Engagement hinsichtlich dieser Thematik vorhanden ist. Damit können wir einen gewissen Bias in den Antworten nicht sicher ausschließen, gehen jedoch davon aus, dass wir trotzdem einen relevanten Querschnitt durch die Versorgungslandschaft von Menschen mit Typ-2-Diabetes und chronischer Nierenerkrankung in Deutschland abbilden konnten.

## Fazit für die Praxis

- Die am häufigsten als relevant erachteten Leitlinien in der Diagnostik und Verlaufsbeobachtung von Patienten mit Diabetes mellitus Typ 2 und chronischen Nierenerkrankungen sind die Praxisempfehlung der Deutschen Diabetes Gesellschaft (DDG) zu Nephropathie bei Diabetes sowie die Nationale VersorgungsLeitlinie Typ-2-Diabetes. Die darin formulierten Empfehlungen stimmen weitgehend mit den internationalen Standards von Kidney Disease: Improving Global Outcomes (KDIGO) und der American Diabetes Association (ADA) überein.
- Gemessen an diesem Standard kann die Messung der geschätzten glomerulären Filtrationsrate in Deutschland als etablierter Standard angesehen werden.
- Klares Verbesserungspotenzial ergibt sich bei den folgenden Punkten:
  Messung der Albuminurie mittels Albumin-Kreatinin-Verhältnis im Urin
  Häufigkeit der Testung
  Zeitpunkt der nephrologischen Konsultation
- Alle Leitlinien und Praxisempfehlungen betonen die interdisziplinäre Versorgung mit einer frühzeitigen, engen und patientenzentrierten Kooperation zwischen Allgemeinmedizin, Diabetologie, Nephrologie und Kardiologie. Die Bildung regionaler Netzwerke kann dazu einen wertvollen Beitrag leisten.

## Footnotes

1 Zeitpunkt der Recherche bis Mai 2023.
